# Size distribution of virus laden droplets from expiratory ejecta of infected subjects

**DOI:** 10.1038/s41598-020-78110-x

**Published:** 2020-12-03

**Authors:** S. Anand, Y. S. Mayya

**Affiliations:** 1 Health Physics Division, Bhabha Atomic Research Centre, Mumbai, 400085 India; 2 Homi Bhabha National Institute, Bhabha Atomic Research Centre, Mumbai, 400094 India; 3 Department of Chemical Engineering, Indian Institute of Technology-Bombay, Mumbai, 400076 India

**Keywords:** Applied mathematics, Scientific data, Influenza virus, Viral infection, Environmental sciences

## Abstract

For rebooting economic activities in the ongoing COVID-19 pandemic scenario, it is important to pay detailed attention to infection transfer mechanisms during interaction of people in enclosed environments. Utmost concern is the possibility of aerosol mediated infection transfer, which is largely governed by the size distributions of virus laden droplets, termed as virusols in this work, ejected from humans. We expand on the well-known theory of Poisson fluctuations which acts as statistical barrier against formation of virusols. Analysis suggests that for viral loads < 2 × 10^5^ RNA copies/mL, often corresponding to mild-to-moderate cases of COVID-19, droplets of diameter < 20 µm at the time of emission (equivalent to ~ 10 µm desiccated residue diameter) are unlikely to be of consequence in carrying infections. Cut-off diameters below which droplets will be practically free of contamination, are presented as a function of viral loading. The median diameters of virus laden polydisperse droplet distributions will be 1.5 to 20 times higher depending upon the geometric standard deviation. The studies have implications to risk assessment as well as residence time estimates of airborne infections in indoor environments. Additionally, it will be also helpful for performance evaluation of sanitization and control technologies to mitigate infection risks in workplaces.

## Introduction

The outbreak of Coronavirus disease (COVID-19) has spread to more than 200 countries in the world, causing global health emergency as the number of confirmed cases reached 45,25,497 including 3,07,395 deaths worldwide as of May 17, 2020^1^. The contagion of COVID-19 is identified as severe acute respiratory syndrome coronavirus 2 (SARS-CoV-2)^2^. Recognized routes^3–5^ of virus transmission from an infected person are, (1) surface/contact transmission, (2) direct droplet transmission, and (3) aerosol transmission. It is presumed that the first two modes of transmission pose the greatest risk, and have formed the backbone of instituting of intervention measures and strategies, such as social distancing, lock down, sanitization, wearing of masks etc. The airborne transmission risk, or the aerosol risk received prominence following a publication by Morawska and Milton^6^, in which special attention was drawn to certain superspreading events such as choir practice, restaurant, etc. As a result, WHO^7^ and CDC^8^ issued scientific brief indicating that airborne transmission is possible under special circumstances like enclosed spaces with inadequate ventilation and prolonged exposure from a severely infected person. Nevertheless, there are many other critical data such as minimum infectious dose for SARS-CoV-2, relationship of disease severity with viral load, proportion of infections acquired through airborne transmission, etc. which need to be known to answer the significance of airborne transmission. The problem is particularly relevant when transfer by severe symptomatic or asymptomatic individuals is considered. In spite of masks which would suppress direct transmission due to sneezing or coughing, it is still possible that viruses from an asymptomatic person might escape into air space through uncontrolled leaks. Recent studies^9,10^ show that the speech droplets are also potential in the virus transmission and the very recent case of COVID-19 outbreak in an air-conditioned restaurant suggests that virus-laden aerosol droplets could have remained in air and travelled long distances before infecting the others^9^.

Several publications^3,4,11,12^ have appeared over the past decade as well as recently on airborne risk, and Tellier et al.^5^ provide an excellent review on the subject. It has been found that significant (42–63%) portion of droplets containing virus causing influenza are in the respirable size range^12–14^ and support the hypothesis that influenza could be transmitted by the airborne route. The potential for airborne risk has a strong implication for the post lock down rebooting of business and office activities. This is because, in enclosed and indoor environments, such as public transport, offices, work places and schools, even a possibility of leaks from ill-fitting masks will be perceived as posing a risk of high consequence and will form a deterrent to a minimal level of interpersonal interaction. This can only be countered by building confidence through the deployment of adequate mitigation/sanitization technologies to stall aerosol route of transmission. The size distribution of airborne contaminants plays a crucial role in their risk potential, inhalability, site of deposition in the respiratory tract, transport in air and removal characteristics by intervention technologies.

The expiratory activities (breathing, speaking, coughing, sneezing, vomiting, etc.) of infected human subjects generate aerosol droplets of different characteristics in terms of their size and initial speed. The airborne droplet with sizes varying from 0.05 to 500 μm^3,11,15^, consist of sub-micron droplets directly emitted due to respiratory activities and the droplet nuclei formed from the evaporation of super-micron droplets contain viruses of size (0.02–0.3) μm^14^. These droplets are formed through atomization process of respiratory fluids (sputum/saliva)^11,15–18^ having a wide range of viral load (10^2^ to 10^11^) copies/mL^19–21^. The droplets contain soluble nonvolatile materials (Na+ , K+ , Cl−, Lactate, Glycoprotein)^22^ up to about 0.71% mole fraction. A rough but reasonable estimate^22^ shows that the respiratory droplet’s initial diameter is reduced by one-half to form droplet nuclei. The droplets of sizes less than about 20 µm (equivalent to ~ 10 µm desiccated residue diameter), which are of importance from airborne risk perspective would dry up within a few seconds to form nonvolatile residues of size approximately half the droplet size^22^. In a recent paper by Stadnytskyi et al.^10^, a factor of 1/3 has also been used for the dehydrated residue sizes. The SARS-CoV-2 virus particles of size (100–200) nm will be incorporated into these residues which will then vector them across the indoor air space.

Another important aspect of size distribution relevant to airborne risk arises from the well-known theory of atomization of suspensions and radioactive aerosol activation mechanisms^23–25^. Due to the discrete nature of the virus (RNA copies), statistical fluctuations become very important for viral incorporation into droplet-residue system during their formation in human ejecta^26^. The studies by Fuchs and Sutugin^23^ and Raabe^24^ showed that particles contained in droplets produced by atomizing suspensions, are distributed according to Poisson distribution which makes allowance for the probability of occurrence of blank droplets with no viral copy. Alonso et al.^27^, in their experimental study indicated that the viral loading is higher in bigger particles than in smaller ones. Fernandez et al.^28^ demonstrated that the probability of number of bacterial cells in an aerosol droplet increases with bacterial solution concentration, and the probability follows Poisson distribution. Zuo et al.^29^ found that the virus loading of droplets follows a power law with exponent > 3, and they showed that the virus laden droplet size distribution shift towards larger particles. Although the work of Shindle and Galily^30^ raised doubts on the Poissonian assumption through their spray drying experiments, the assumption is still widely used for want of an alternative formulation. In a very recent work, Madas et al.^31^ have used Poisson distribution to estimate the probability of an airborne particle carrying at least one virus copy for modeling the deposition distribution of the SARS-CoV-2 virus in the human airways. As a result of the fluctuations, significant part of the ejected droplets would dry up to form blank residues carrying no RNA copies, thereby becoming unviable and harmless from the point of view of infection transfer. This situation is at variance with the assumption of virus distribution in the aerosol as being “proportional to droplet volume”^4,17^.

Stadnytskyi et al.^10^ estimated that for viral load of 7 × 10^6^ RNA copies/mL, less than 0.01% of 3 µm, 0.37% of 10 µm, 37% of 50 µm droplets (prior to dehydration) will carry one or more virus and the remaining fraction will not carry any virus. Although they did not mention the basis of their calculations, a quick comparison with the formula [Eq. (3)] in this paper confirms that they have made use of Poisson fluctuations for their estimates. For polydisperse droplets, the size dependent nature of the Poisson incorporation probabilities renders the size distribution of droplets carrying virions at variance with that of the original droplet (or residue) size distributions. In view of the huge significance of the infection carrying droplets and particles during a pandemic, it may be useful to distinguish them from normal aerosols by a separate nomenclature, and we suggest a terminology, “virusols” to convey virus incorporation. We feel that this coinage will help in focussing on the virus-laden, rather than generic, aerosol size spectrum. The purpose of this note is to provide quantitative estimates of the salient distinguishing features of the virusol systems as a function of an appropriately defined propensity parameter. This provides backup rationale for the assessment of airborne risk as a function of emitted viral load from the infected person.

## Methods

If ${C}_{v}$ is the average concentration (RNA copies/mL) of the virus in the biological fluids/samples (sputum/saliva/respiratory fluids), then the strength of incorporation into a droplet of diameter (${d}_{p}$) will be proportional to the mean expected number ($\mu$) of the viral copies in the droplet, expressed as

1 $$\mu =\frac{\pi }{6}{d}_{p}^{3}{C}_{v}$$

The quantity $\mu$ may also be recognized as the “propensity parameter” for the formation of the virus-laden particles, or virusols. The probability ${P}_{n}$ that a droplet will actually contain *n* viral copies follows from the one parameter Poisson distribution, having mean $\mu$ and standard deviation $\sqrt{\upmu }$:

2 $${P}_{n}=\frac{{\left(\mu \right)}^{n} exp\left(-\mu \right)}{n!}$$

whence it follows that the probability of containing no virus at all ($n=0$) will be ${e}^{-\mu }$.

It may be remarked here that Buonanno et al.^32^ introduced the concept of Poisson fluctuations at the level of exposure calculation post-inhalation of droplet from an infected person. This is different from assigning Poisson fluctuations at the level of droplets as we have done here which we believe, possesses greater versatility for developing risk transfer models by making allowance for potential fluctuations in the number of inhaled droplets. The complementary probability that the droplet will contain at least one virus will be the probability of formation of a virusol, given by

3 $${P}_{v}=1-{e}^{-\mu }$$

From Eq. (3), it is seen that *P*_*v*_, the fraction of virus-laden droplets, would be closer to unity (i.e. all ejected droplets are contaminated) only when propensity parameter exceeds the order of unity. In most practical situations of interest to airborne infections, this would be unlikely, as may be seen below.

The viral load in the infectious subjects varies over a wide range due to many factors and infection time^11,19,20^. Table 1 shows compilation of viral load data from the literature. Most of the data comes from recent studies associated with SARS-CoV-2 and hence have significant topical relevance. The study of Zheng et al.^33^ is comprehensive and shows a broad range between (10^2^–10^7^) RNA copies/mL. There is a general conformity between all the reported data regarding the range, and an atypical high value of 1.3 × 10^11^ RNA copies/mL is reported by Pan et al.^21^ from sputum sample of a patient. The propensity parameter corresponding to ~ 10^8^ RNA copies/mL will be close to unity for a droplet diameter of ~ 20 µm. One can expect that droplets below this size will be increasingly blank.

**Table 1:** Virus load in various biological fluids among various categories.

References	Number/Type of samples	Number of individuals and category	Median Concentration, copies/mL	Remarks
Hirose et al. (2016)^19^	–	22	Sputum—2.4 × 10^7^ (mean value)	Range—8.9 × 10^4^–2.7 × 10^8^ copies/mL
To et al. (2020)^20^	173 samples	23		Range—10^3^–3.2 × 10^7^ copies/mL Initial concentration of 3 patients were 10 copies/mL
		13—mild	Initial—1.3 × 10^5^ Peak—2.0 × 10^5^	
		10—severe	Initial—1.5 × 10^6^ Peak—8.1 × 10^6^	
Pan et al. (2020)^21^	110 samples	80	Throat—7.6 × 10^4^ Sputum-7.52 × 10^5^	Range—6.4 × 10^2^–1.3 × 10^11^ copies/mL
Zheng et al. (2020)^33^	1846 respiratory samples (sputum & saliva)	96		Range—10^2^–10^7^ copies/mL
		22—mild	10^4^	
		74—severe	10^5^	
Zou et al. (2020)^35^	Nasal and throat samples	18 1—asymptomatic 3—severe 14—mild-to-moderate	Peak—~ 10^8^	Asymptomatic case (Nasal—~ 10^5^–10^7^ copies/mL; Throat—~ 10^4^ copies/mL)
Wolfel et al. (2020)^39^	Sputum samples	9	7 × 10^6^	Maximum–2.4 × 10^9^ copies/mL

Given the sparsity of data and the fact that the numbers would vary from patient to patient, and from time to time, no empirical correlation seems to have been established between viral concentrations and the severity of symptoms. In a recent study, Liu et al.^34^ concluded that severe cases have distinctly higher viral loading as compared to milder cases. Also, from Table 1, it is clear that on an average, patients with severe symptoms show higher viral load as compared to patients with mild symptoms^20,33^. Broadly speaking, mild cases fall in the category median of < 2 × 10^5^ RNA copies/mL as compared to the levels of > 10^6^ RNA copies/mL for those with severe symptoms^20,34^. With this empirical understanding, we tentatively classify viral load into the following two categories:

- Mild-to-moderate cases: ${{10}^{2}/mL<C}_{v}<2\times {10}^{5}/mL$
- Severe cases: $${C}_{v}>{2\times 10}^{5}/mL$$

In a few recent studies^35,36^, asymptomatic cases have been found to carry viral load similar to mild cases with one case of exceptionally high load (Table 1). We presume that this is an unlikely scenario in general, and consider asymptomatic situation as falling within mild-to-moderate loading. It may be noted that severe cases are very unlikely to be found in public indoor spaces, as they are likely to have gone for medical attention or quarantining.

The data on droplet size distributions, obtained in a few earlier studies in respect of ejecta droplets is presented in Tables 2 and 3. While Table 2 consists of information on mean sizes, and geometric standard deviations, Table 3 is a special study by Morawska et al.^37^ in which droplet concentration data is presented for a few size classes.

**Table 2:** Lognormal size distribution data.

References	Remarks	Count median diameter (CMD)/geometric mean (GM), μm	Geometric standard deviation (GSD)	Total number/number concentration
Lindsley et al. (2012)^12^	Unimodal fit	CMD—0.63 VMD—2.44	1.54–1.83 1.66–2.31	16.8–29.6 # cm^−3^
Nicas et al. (2005)^22^	Duguid’s cough data	GM—14	2.6	5 × 10^3^ #
	Duguid’s sneeze data	GM—8.1	2.3	1 × 10^6^ #
	Loudon and Roberts’s cough data—unimodal fit	GM—24	8.4	4.7 × 10^2^ #
	Loudon and Roberts’s cough data—bimodal fit	GM1—9.8 (71%) GM2—160 (29%)	GSD1—9 GSD2—1.7	4.7 × 10^2^ #
Johnson et al. (2011)^40^	Trimodal distribution	CMD1–1.6; CMD2–1.7; CMD3–123	GSD1—1.25; GSD2—1.68; GSD3—1.84	Cn1–0.09 # cm^−3^; Cn2–0.12 # cm^−3^; Cn3–0.02 # cm^−3^; Total—0.22 # cm^−3^

**Table 3:** Droplet size distribution data for different expiratory activities from Morawska et al. (2009)^37^.

Mid-point droplet diameter, μm	Droplet number concentration, # cm^-3^			
	Speaking	Breathing	Whispered counting	Voice counting
0.8	0.751	0.084	0.236	0.110
1.8	0.139	0.009	0.068	0.014
3.5	0.139	0.003	0.007	0.004
5.5	0.059	0.002	0.011	0.002

## Results and discussion

The results presented here consider the cases of airborne droplets prior to evaporation, just released due to expiratory processes such as coughing, speaking, etc. Due to inevitable evaporation process, the droplet diameters are reduced to ~ 50% of their original value^38^ in a very short time (in few seconds and less). For all practical purposes, a susceptible person will be exposed to inhalation of dried droplets. In view of the fact that virusols > 10 µm are unlikely to reach the pulmonary region, and cause risks, they are not considered from the risk perspectives^22^. From the droplet perspective, this amounts to a cut-off diameter of 20 µm and it’s required to examine the virus carrying potential (virusol potential) of droplets lower than 20 µm.

**Figure 1:**
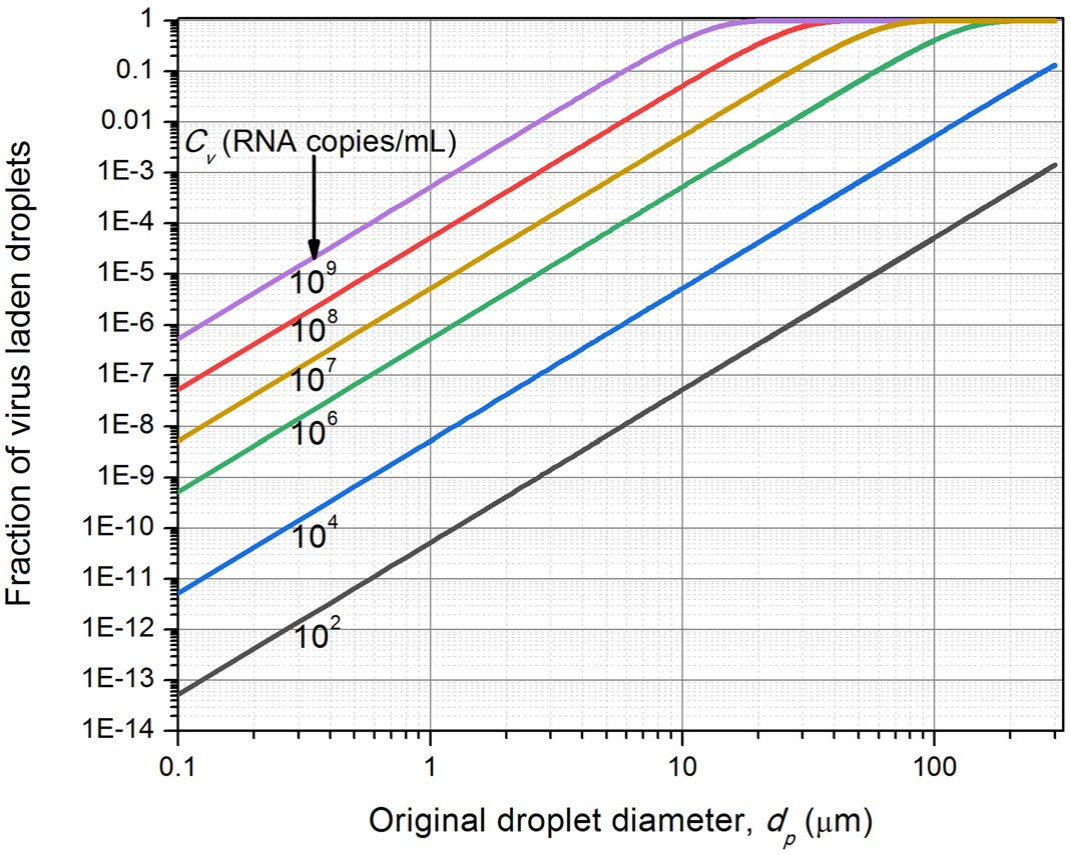
Fraction of virus-laden droplets formed from the ejected droplets, as a function of its size and viral load in the fluid.

Figure 1 shows a graphical representation of Eqs. (1) and (2) for the variation of the virus laden droplet (virusol) fractions with respect to the emitted droplet diameter for different viral loading, which includes the cases of Stadnytskyi et al.^10^. Relationship between viral loading and severity of the disease is a matter of considerable practical value. For viral loads of less than 10^4^ RNA copies/mL, expected for mostly mild-to-moderate cases^21,33^, the virusol fraction is less than 0.1% for droplets below 60 µm; i.e. more than 99.9% of the droplets below 60 µm will not be carrying any virus. As larger than 60 µm droplets are very unlikely to remain airborne for infecting via aerosol route, this simple analysis leads to a conclusion that mild-to-moderate cases are least likely to infect via aerosol route.

**Figure 2:**
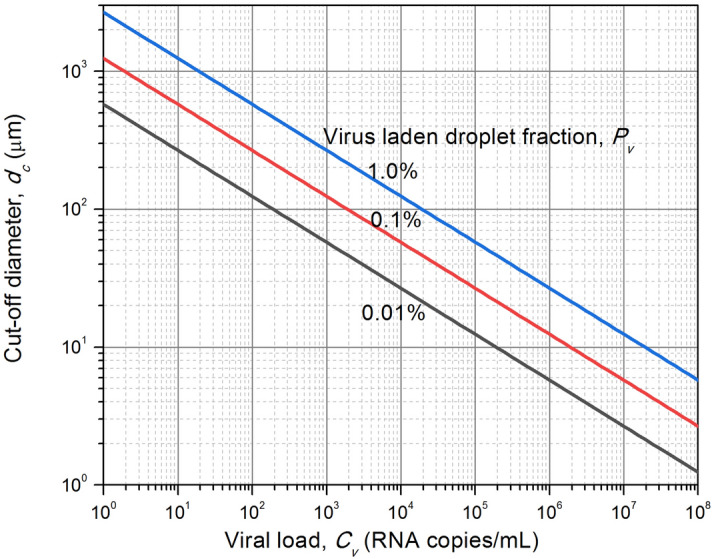
Smallest droplet diameter likely to be contaminated as a function of viral load in ejecta.

Figure 2 provides a summary representation of the viral load dependence of the cut-off diameters below which the virus contaminated fractions of droplets will be lower than 0.01%, 0.1% and 1% respectively. From Tables 2 and 3, one may infer that if the total ejected droplets per forced ejection event that are likely to leak out from masks and remain suspended in air, to be less than about 1000, then a level of 0.1% or less should be sufficiently safe as it would imply less than about 1 virus carrying droplet per ejection event. From an infected personnel risk point of view, it appears from Fig. 2 that for mild-to-moderate cases with viral loading around 2 × 10^5^ RNA copies/mL, droplets less than 20 µm are unlikely to carry any viral load. Thus, airborne contamination is most likely to arise from severe patients only. The figures also show that even for ejections from infected subjects with high viral load, the droplets < 2 μm (prior to evaporative water loss) are unlikely to be contaminated and carry no risk. From Table 3, it is seen that most of the particles are generated in the size range of (0.8–1.8) μm for the expiratory events such as speaking, singing, breathing, etc. with a maximum number concentration of ~ 1 cm^−3^ (for speaking). The corresponding virus laden fraction (virusols) are in the range of 2 × 10^−5^–4 × 10^−4^ even for severe cases with viral load of 10^8^ RNA copies/mL. In real indoor scenario, this would amount to risk of inhaling less than one virus carrying droplet if a person stays for an hour in the room with the infected person. One can therefore restrict attention on large droplets only for aerosolized risks, and the remaining fraction will be just uncontaminated droplets.

The above arguments have a significant implication on the virusol size distributions in polydisperse droplet systems. Most of the aerosol droplet’s size measurements, carried out with optical sizing instruments, are fitted to lognormal distributions. The total aerosolized droplet concentration varies over a wide range from ~ 1 cm^−3^ to 2.5 × 10^3^ cm^−3^ (Tables 2, 3). It must be admitted that the complete size distribution data are rather sparse, especially on smaller droplets and further, no recent data from the ongoing pandemic situation, is available. The presented data (Tables 2, 3) from different research groups show large variability in terms of number of modes, median sizes as well as extent of dispersity. In the < 20 μm size mode which is of interest from airborne point of view, the modal values vary from 0.63 to 24 μm. There is also large variation in geometric standard deviations (GSDs) and unusually high value of about 8.4 and 9 are also reported. GSD values more than 4 are generally exceptional, those beyond 8 may not be acceptable as they would give rise to unphysical mass content from the given number of droplets^22^. We thus ignore these cases and limit our analysis to distributions up to GSD = 4 only.

For a lognormal distribution of aerosol droplets with volume median diameter (VMD, ${d}_{G}$) (which is also geometric mean volume diameter) having geometric standard deviation ${\sigma }_{G}$, the fraction (${F}_{v}$) of droplets laden with at least one virus, is given by,

4 $${F}_{v}=\frac{1}{\sqrt{2 \pi } \mathrm{ln}{\sigma }_{G}} {\int }_{0}^{\infty }\left[1-exp\left(-\frac{\pi }{6}{d}_{p}^{3} {C}_{v}\right)\right] exp\left(-\frac{{\left\{\mathrm{ln}\frac{{d}_{p}}{{d}_{G}}\right\}}^{2}}{2{\mathrm{ln}}^{2}{\sigma }_{G}}\right) \frac{d{d}_{p}}{{d}_{p}}$$

**Figure 3:**
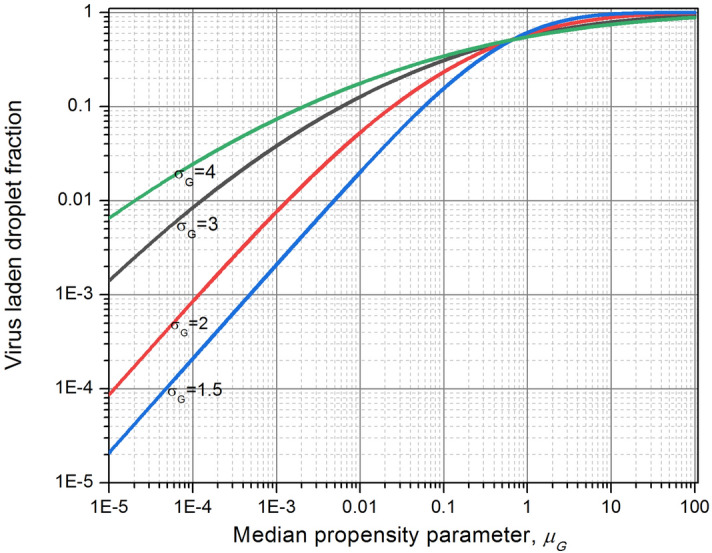
Virusol fraction of lognormally distributed ejecta droplets as a function of viral load in patients.

In analogy with radioactive tagging of aerosols, we can consider the present condition as viral tagging of droplets, or as proposed in the introduction, as virusols. By using Eq. (4), results are presented in Fig. 3 for different GSDs between 1.5 and 4. Figure 3 shows the variation of virusol fraction with respect to the median propensity parameter, ${\mu }_{G}$ defined as, $\frac{\pi }{6}{d}_{G}^{3} {C}_{v}$.

**Figure 4:**
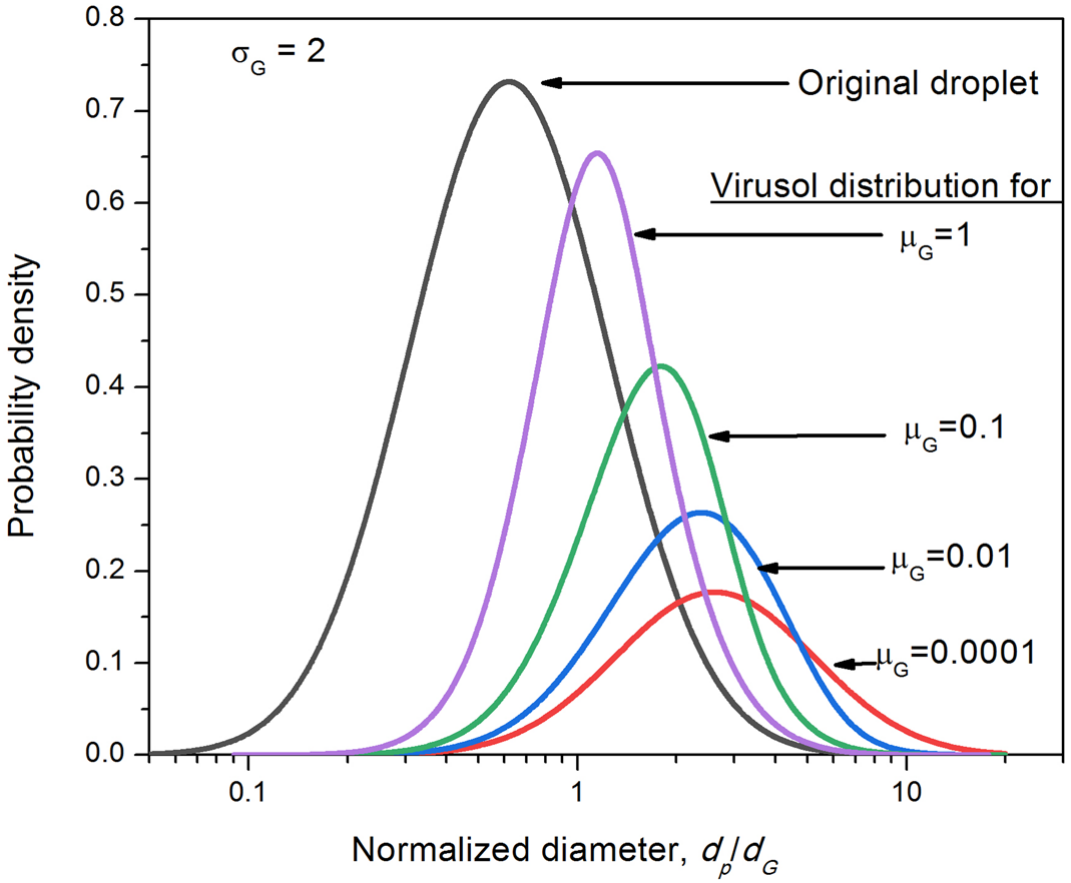
Virusol size-distribution for different propensity parameter, ${\mu }_{G}=\frac{\pi }{6}{d}_{G}^{3} {C}_{v}$.

As seen in Fig. 3, less than 10% of the droplet spectrum is contaminated for ${\mu }_{G}$ < 0.005 which would cover all droplets below 20 μm size from mild-to-moderate patients (${\sigma }_{G}=3$). The graph shows an interesting cross over point at ${\mu }_{G}$ = 0.6 wherein virusol fraction is 50% regardless of ${\sigma }_{G}$ (This point varies between 0.58 and 0.62). Most of the measured GM and GSD data fall within the data domain for which viral contamination probability is less than 1%. Figure 4 shows the normalized virusol size distributions as contrasted from the original airborne droplets, for various viral loads and droplet mean size, captured by a single propensity parameter $\left({\mu }_{G}\right)$. Plots in Fig. 4 clearly shows a distinct shift in the virusol mode as compared to droplet aerosols for ${\sigma }_{G}=2$. The shift will be more pronounced for higher ${\sigma }_{G}$. The plots illustrate how the lower end of the size spectrum, which will contain large proportion of droplets, is hardly contaminated by virus.

**Figure 5:**
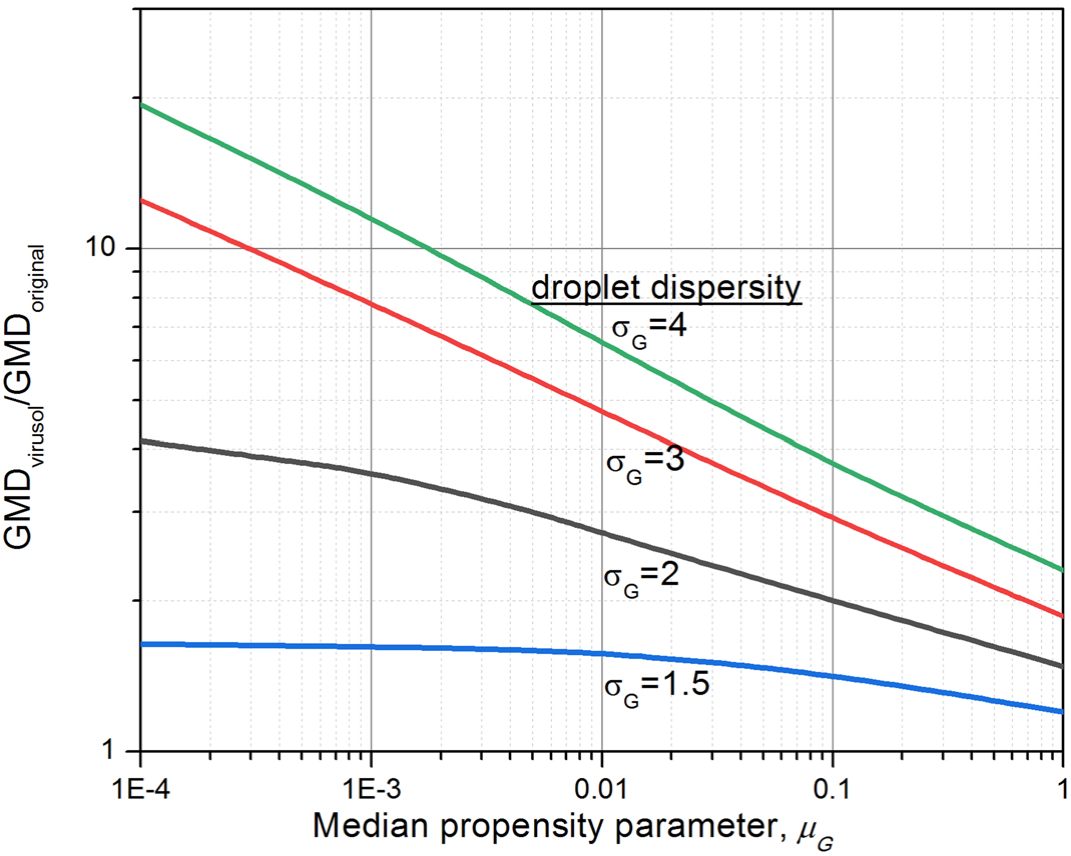
Variation of median size of virus-laden droplets (Virusols) relative to the original droplets with respect to propensity parameter $\left({\mu }_{G}\right)$ for different dispersity measure (${\sigma }_{G}$ of droplets).

Finally, it would be useful to provide a prescription to convert droplet size distribution to virus-laden droplet distribution. It must be admitted that the virusol distribution does not strictly satisfy lognormal form even if the droplet aerosols are lognormally distributed: the deviation is higher for higher propensities. Nevertheless, one can fit a single mode lognormal to obtain the set of virusol parameters that would reflect the median size and dispersity parameters to a good approximation. This is achieved by conducting a series of apparent linear fits to the cumulative lognormal data plotted on log-probability graph. The results are presented in Fig. 5, which shows the variation of the ratio of the VMD of virusol and that of the original droplet, as a function of the median propensity parameter ${\mu }_{G}$ for different ${\sigma }_{G}$. Increasing ${\sigma }_{G}$ brings in larger enhancement of the median size of the virusol. The enhancement could be by a factor of 1.5 to 20 in the range of interest indicated. It is found that for the data in the figure, the GSD values remain almost constant. For the droplet ${\sigma }_{G}$ values of 1.5, 2, 3, and 4, the GSD of virusol distribution lies in the range of 1.48–1.52, 1.75–1.85, 2.25–2.5, and 2.2–3.3 respectively. Because of a “statistical barrier” against viral incorporation into smaller droplets, the ${\sigma }_{G}$ of the virusol systems seem to be smaller than that of the original droplet systems.

## Conclusions

The virus-laden droplets of sizes of about 20 μm (equivalent to ~ 10 µm desiccated residue diameter) or less ejected from human ejecta of infected persons are matter of potential concern from the hazard perspective of viral transmission by airborne route in confined environments. As we know, not all droplets carry viruses and this fact has bearing on future intervention technologies which operate on size control basis. An important “statistical barrier” brought about by Poisson fluctuations limits viral incorporation into droplets during their ejection from the infected subjects. The present analysis illustrates the impact of this reasoning on the formation of virus laden droplet systems, termed herein as virusols, their size distributions and practically useful cut-off values. By combining the available data from the current literature on viral loading in different patients with the recent observation on its relationship with disease severity, it is argued that formation of virusols, which will remain stable for certain length of time as well as which are inhalable by humans, (i.e. droplets less than 20 μm) is virtually inhibited in mild-to-moderate cases of patients. Virusol formation and consequent infection transfer could be important for explicitly severe cases, that too for droplet sizes above 2 µm (prior to evaporation). Hence, for an effective control measure using filtration based air cleaners, it may not be necessary to worry about ultrafine particle filtration. This somewhat relaxes the constraint on the filtration efficiency as relatively coarser filters will be efficient in capturing larger particles. As a result, flow resistances can be significantly lowered thereby enabling higher Clean Air Delivery Rates. Furthermore, the finding of a significant upward shift in virusol sizes, implies that their residence times in indoor spaces will be considerably lower than other droplets ejected from humans. This will greatly help in providing a realistic assessment of air borne infection transfers in indoor environments.
